# Applying Advanced Imaging Techniques to a Murine Model of Orthotopic Osteosarcoma

**DOI:** 10.3389/fsurg.2015.00036

**Published:** 2015-08-03

**Authors:** Matthew L. Broadhead, Zerina Lokmic, Mei Lin Tan, Andrew Stevenson, David S. Binns, Carleen Cullinane, Rodney J. Hicks, Peter F. M. Choong, Damian E. Myers

**Affiliations:** ^1^Department of Surgery, St. Vincent’s Hospital Melbourne, University of Melbourne, Fitzroy, VIC, Australia; ^2^Vascular Biology Laboratory, Murdoch Children’s Research Institute, Parkville, VIC, Australia; ^3^Materials Science and Engineering, CSIRO, Clayton, VIC, Australia; ^4^Peter MacCallum Cancer Centre, East Melbourne, VIC, Australia

**Keywords:** osteosarcoma, luminescent measurements, x-ray microtomography, positron-emission tomography, pigment epithelium-derived factor

## Abstract

**Introduction:**

Reliable animal models are required to evaluate novel treatments for osteosarcoma. In this study, the aim was to implement advanced imaging techniques in a murine model of orthotopic osteosarcoma to improve disease modeling and the assessment of primary and metastatic disease.

**Materials and methods:**

Intra-tibial injection of luciferase-tagged OPGR80 murine osteosarcoma cells was performed in Balb/c nude mice. Treatment agent [pigment epithelium-derived factor (PEDF)] was delivered to the peritoneal cavity. Primary tumors and metastases were evaluated by *in vivo* bioluminescent assays, micro-computed tomography, [^18^F]-Fluoride-PET and [^18^F]-FDG-PET.

**Results:**

[^18^F]-Fluoride-PET was more sensitive than [^18^F]-FDG-PET for detecting early disease. Both [^18^F]-Fluoride-PET and [^18^F]-FDG-PET showed progressive disease in the model, with fourfold and twofold increases in standardized uptake value (*p* < 0.05) by the study endpoint, respectively. *In vivo* bioluminescent assay showed that systemically delivered PEDF inhibited growth of primary osteosarcoma.

**Discussion:**

Application of [^18^F]-Fluoride-PET and [^18^F]-FDG-PET to an established murine model of orthotopic osteosarcoma has improved the assessment of disease. The use of targeted imaging should prove beneficial for the evaluation of new approaches to osteosarcoma therapy.

## Introduction

Osteosarcoma is the most common primary malignancy of bone and the second most common cause of cancer-related death in the pediatric age group (1). Patients treated with surgical resection and three-drug chemotherapy regimes have an overall 5-year survival rate of 70%. Patients who do not respond to multi-agent chemotherapy have limited treatment options. Intensification of treatment or substitution of chemotherapeutic agents has not proven to be of benefit to these patients (2, 3).

Osteosarcoma cells metastasize to the lungs and a number of molecular pathways are being targeted in the development of novel treatment agents. In order to evaluate these novel treatment agents, it is critical that preclinical translational studies are performed using reproducible animal models that closely simulate human osteosarcoma.

Pigment epithelium-derived factor (PEDF) is a glycoprotein that has shown promise in early laboratory studies as a targeted anti-osteosarcoma agent. PEDF is an anti-angiogenic endogenous glycoprotein that simultaneously targets osteosarcoma cells and inhibits tumor vessel formation (4). *In vivo* studies evaluating PEDF therapy have made use of a clinically relevant murine model of orthotopic osteosarcoma. This model phenocopies the pathological features and behaviors of human osteosarcoma (5). When an intra-peritoneal micro-osmotic pump delivered PEDF systemically, the growth of well-established orthotopic osteosarcoma was suppressed, and the burden of pulmonary metastatic disease was reduced (6).

A key aim in this study was to improve the murine model of orthotopic osteosarcoma by making use of advanced imaging techniques; bioluminescence imaging, micro-computed tomography, [^18^F]-Fluoride-PET and [^18^F]-FDG-PET. We sought to evaluate whether these imaging techniques could be used to provide well-defined and reproducible endpoints for evaluating potential therapies, such as PEDF, in the murine orthotopic model.

## Materials and Methods

### Osteosarcoma cell line

The luciferase-expressing OPGR80 cell line was derived from a transgenic mouse model of conditional osteosarcoma. The cell line was kindly donated by Dr. Carl Walkley (St. Vincent’s Institute, Melbourne, VIC, Australia). In this model, osteoblast-restricted deletion of p53 and pRb generate spontaneous multifocal osteosarcoma (7). The luciferase-containing vector was kindly provided by Dr. Andrew Kung (Dana-Farber Cancer Institute, Boston, MA, USA). Luciferase and phosphotransferase sequences were fused and introduced into a pMMP retrovirus to generate pMMP-LucNeo. Cells were co-transfected with pMMP-LucNeo and Eco Pac plasmid. Viral supernatants were then applied to the OPGR80 cell line for 48 h. Luciferase-expressing cells were selected with 1 mg/mL neomycin (G418, Invitrogen, Carlsbad, CA, USA).

OPGR80 cells were cultured under standard conditions of 37°C/5% CO_2_ in complete medium (CM), which consisted of MEM-Alpha + GlutaMAX (Invitrogen, Carlsbad, CA, USA) supplemented with 10% fetal bovine serum (Invitrogen, Carlsbad, CA, USA) and 1% antibiotic–antimycotic (Invitrogen, Carlsbad, CA, USA).

### MTS proliferation assay

OPGR80 cells were seeded in 96-well plates in CM, at a density of 1 × 10^3^ cells/well, 100 μL/well. Cells were cultured under standard conditions for 24 h and then treated with PEDF at 0, 1.56, 3.125, 6.25, 12.5, 25, 50, and 100 nM concentrations in quadruplicate. After 48 h of exposure to the relevant treatment, 20 μL of CellTiter 96^®^ AQ_ueous_ One Solution Reagent (Promega, Madison, WI, USA) was added to each well. This reagent contains the tetrazolium compound MTS (3-(4,5-dimethylthiazol-2-yl)-5-(3-carboxymethoxyphenyl)-2-(4-sulfophenyl)-2H-tetrazolium) that is bioreduced into a colored formazan. Cells were incubated with the reagent for 3 h and absorbance was measured each hour at 490/655 nm using a Bio-Rad Model 680 micro-plate reader (Bio-Rad, Philadelphia, PA, USA).

### Animals and ethics approval

Animal Ethics Committees at St Vincent’s Hospital Melbourne (033/10, 034/10) and Peter MacCallum Cancer Centre (E461) approved all animal experimentations. Five-week-old Balb/c nude mice were purchased from the Animal Resource Centre Australia.

Mice were kept in polycarbonate micro-isolator cages with individual air supplies and temperature was maintained at 23°C, with a 12-h light–dark cycle. Animals were handled at all times in a laminar flow hood and were observed for behavioral changes, such as abnormal grooming and foraging, inactivity, and lameness. Mice were weighed using digital scales and were monitored thrice weekly for tumor growth and signs of distress. Tumors were measured in the anteroposterior (AP) and lateral (L) planes using digital calipers. Leg volume was calculated using the formula 4/3π[1/4(AP + L))]^2^. The volume of the contralateral limb was subtracted from that of the tumor-bearing limb to calculate tumor volume, as previously described (6, 8).

### Intra-tibial inoculation of OPGR80 osteosarcoma cells

Luciferase expression by OPGR80 cells was confirmed *in vitro* prior to performing intra-tibial inoculations. OPGR80 cells were seeded in duplicate in a 96-well opaque white Microtest™ plate (BD Biosciences, Franklin Lakes, NJ, USA) 100 μL/well, at concentrations of 2.5 × 10^5^, 1.25 × 10^5^, 6.25 × 10^4^, and 3.125 × 10^4^ cells/well. Cells were then incubated for 6 h under standard conditions before the addition of 150 μg/mL d-luciferin (Caliper LifeSciences, MA, USA). An *in vitro* bioluminescent assay was then performed by centering the plate on the imaging stage of the Xenogen IVIS^®^ Spectrum imaging system. Total flux for each well was measured using the auto-exposure and auto-region of interest (ROI) functions of Living Image^®^ software (Caliper LifeSciences, MA, USA).

OPGR80 cell viability was confirmed by Trypan Blue exclusion immediately prior to intra-tibial inoculation. Cells were then mixed with 50% BD Matrigel™ Matrix (BD Biosciences, Franklin Lakes, NJ, USA) to a concentration of 2 × 10^6^ cells/mL. BD Ultra-Fine Insulin Syringes (BD Biosciences, Franklin Lakes, NJ, USA) were filled with 10 μL of the OPGR80/Matrigel solution and kept on ice. Ten microliters of intra-tibial injections were performed, as previously described by Dass et al. (5). Mice were anesthetized with intra-peritoneal ketamine (100 mg/kg) and xylazine (10 mg/mg). The left leg of each mouse was held with the knee maximally flexed and the 29G needle tip was placed at the tibial tuberosity. The needle was advanced using a drilling motion to avoid fracture of the bone. The 10 μL inoculum was then injected and the needle retracted slowly to avoid back-flow.

### Systemic delivery of treatment agents

The day of orthotopic injection of osteosarcoma inoculum was defined as day 0 of the study. Treatment was initiated at day 20 of the study as per previous studies (6). The treatment groups were as follows: (1) a control group of untreated tumor-bearing animals (*n* = 6); (2) a group receiving sterile water as the vehicle by micro-osmotic pump (*n* = 10); (3) a group receiving PEDF by micro-osmotic pump (*n* = 10) at a continuous rate of 500 μg/kg/day. Water was used as the PEDF diluent. Previous experience with this method of PEDF delivery guided selection of the 500 μg/kg/day PEDF dose (6, 8).

### Bioluminescent assay *In vivo*

*In vivo* bioluminescent assays using the Xenogen IVIS were performed at days 3, 6, 10, and 13 following intra-tibial injection of the OPGR80 cell line. Tumor growth was determined by measuring total flux of the primary tumors at each time point. Following implantation of the micro-osmotic pump for PEDF delivery, *in vivo* bioluminescent assays were performed at days 20, 24, 27, and 31.

Ten minutes prior to imaging, mice were given an intra-peritoneal injection of d-luciferin (150 mg/kg, 15 mg/mL, Caliper LifeSciences, MA, USA). Mice were then anesthetized with isoflurane/oxygen (5% induction, 2% maintenance) and were imaged prone using the Xenogen IVIS^®^ Spectrum imaging system. Exposure times varied from 0.5 to 60 s.

### Positron-emission tomography *In vivo*

Positron-emission tomography (PET) imaging was performed *in vivo* utilizing [^18^F]-Fluoride and [^18^F]-fluorodeoxyglucose (FDG) (Peter MacCallum Cancer Centre, East Melbourne, VIC, Australia). Imaging with [^18^F]-Fluoride and [^18^F]-FDG were performed on sequential days, 1 week after intra-tibial injection (*n* = 6) and then at the study endpoint (*n* = 26).

For [^18^F]-FDG-PET imaging, mice fasted for at least 3 h were anesthetized using 3% isoflurane in 50% oxygen in air and then injected intravenously via the tail vein with 14.8 MBq [^18^F]-FDG. Anesthesia was maintained for a further 20 min before the animals were allowed to recover. At 90 min after tracer injection, the mice were anesthetized again before being placed on the bed of a Philips Mosaic PET scanner and imaged over 10 min. For [^18^F]-Fluoride-PET imaging, unanesthetized animals were injected with 14.8 MBq [^18^F]-Fluoride and PET imaging performed 60 min later, as described above.

Positron-emission tomography scans were corrected for decay and random emissions before the images were reconstructed using a 3D RAMLA algorithm. In-house analysis software (Marvn2.01) was used for analysis and interpretation. A blinded single observer defined the volume of interest at the site of the primary tumor and calculated the maximum standardized uptake value (SUV_max_), target to background ratio (TBR), percentage of injected dose (% ID), and metabolic tumor volume (MTV).

### Experimental endpoint and tissue processing

Whole body perfusion with 4% paraformaldehyde (PFA) was performed at the humane endpoint of the study. Animals were first anesthetized with isoflurane using a nose cone. Dissection through the anterior abdominal and thoracic walls was performed to reveal the heart. The infusion apparatus consisted of a Harvard Apparatus Syringe Pump S11 Plus (loaded with a 10 mL syringe containing 4% PFA), connected to a 26G needle by 0.4 mm polyethylene tubing. The needle was introduced into the left ventricle of the heart and 2 mL of 4% PFA was infused systemically, at a rate of 9.49 mL/h.

### Micro-computed tomography post-mortem

Micro-computed tomography was performed post-mortem using the SkyScan system (Bruker microCT, Belgium). Scans were performed of the pelvis, femurs, and tibiae of non-dissected mice using a 0.5 mm aluminum filter, 50 kV voltage, and 98 μA current. 3D volumetric reconstruction was performed using the SkyScan software NRecon (Caliper LifeSciences, MA, USA). Reconstructed slices were viewed using SkyScan software CT-Analyzer. Image segmentation was performed manually to define the radio-dense tumor mass as the ROI. Adjacent cortical bone of the tibia was excluded from the ROI. Tumor volume, radio-dense tumor volume, and % radio-dense tumor were calculated, along with tumor nodule number, separation, and thickness.

### Tissue histology

Tissues from six animals from each treatment group were processed into paraffin blocks for histological examination. These six animals had a primary tumor total flux (obtained at the study endpoint using the Xenogen IVIS^®^ Spectrum imaging system) closest to the median result for each treatment group. Orthotopic tumors were dissected from the adjacent bone post-mortem and prepared for histological examination thus avoiding need to decalcify tumor tissues prior to cutting.

Four-micron-thick sections of primary tumor, lung, heart, kidney, and liver were sectioned to achieve the greatest cross-sectional area for examination. Three random sections, each separated by 50 μm distance in the sample, were mounted on a slide for evaluation. All tissues were stained with hematoxylin and eosin to visualize tissue structure.

To detect tissue calcification, the primary tumor and lung tissues were stained with Alizarin Red S, an anthraquinone derivative, to detect calcium-Alizarin Red S complexes, which are birefringent under polarized light (9). Briefly, the sections were immersed in Alizarin red S for 5 min, excess stain blotted prior to immersion into 100% acetone (20 dips), followed by 50% acetone/xylene (20 dips) and 100% xylene solution (20 dips). The sections were then cover-slipped in DPX and examined by light and polarized microscopy.

Dewaxed primary tumor and lung tissues were stained with indirect immunohistochemistry for CD31 and VEGF-A using an autostainer (DAKO Autostainer, Botany, NSW, Australia). Briefly, dewaxed sections were pre-treated with proteinase K for 8 min (to detect CD31) or 0.1M citric acid buffer pH 6.0 (to detect VEGF-A), followed by blocking of endogenous tissue peroxidase by 3% hydrogen peroxide, three washes in tris-buffered saline (TBS) pH 7.4 and 30 min incubation in protein blocking solution (DAKO, Carpinteria, CA, USA). The sections were then subjected to 1-h incubation in purified rat anti-mouse anti-CD31 antibody (BD Pharmingen, clone MEC 13.3, 1:100 dilution) or rabbit anti-human polyclonal anti-VEGF (Abcam, Cambridge, UK, ab 46154, 1:100 dilution). The unbound primary antibody was washed away with TBS, followed by incubation in secondary polyclonal biotinylated rabbit anti-rat IgG and swine anti-rabbit IgG conjugated to HRP (both purchased from DAKO), to detect CD31 and VEGF-A, respectively. To detect CD31 bound complex ABC Elite was used (Vector laboratories, Burlingame, CA, USA), whereas VEGF-A complex was detected by streptavidin-HRP (DAKO). Bound complexes were visualized by diaminobenzidine (DAKO).

All stained sections were scanned and photographed using an Olympus BX51 microscope, VS120 slide scanner, and Olympus VS-ASW software version 2.4. To examine if there was a difference in the extent of tumor calcification between the experimental groups, the Alizarin Red S staining was quantified from scanned images using ImageJ software version 1.47a (National Institutes of Health, USA) and expressed as percentage of total tumor tissue examined.

### Statistics

GraphPad Prism for Mac OS X (Version 5.0d) was used for all statistics. Student’s *t*-test and ANOVA analysis with Bonferroni multiple comparisons test were used where appropriate. Data were expressed as mean ± SEM, with *p* < 0.05 considered statistically significant.

## Results

### PEDF inhibits proliferation of OPGR80 cells *In vitro*

**Figure 1:**
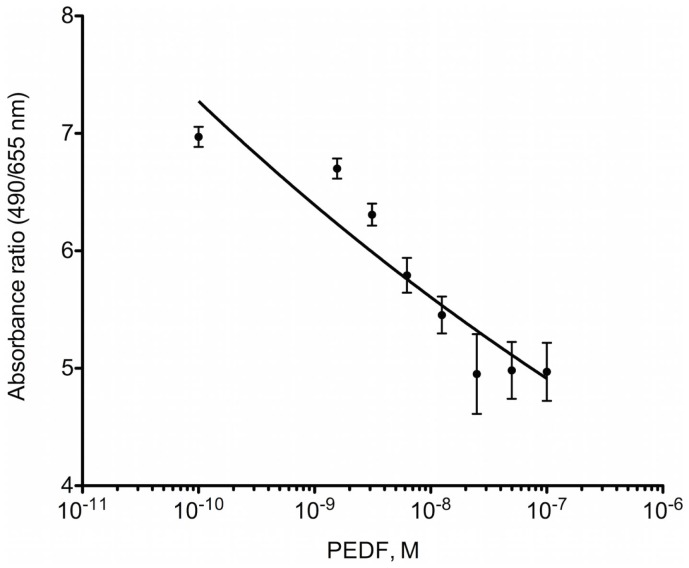
**MTS proliferation assay**. OPGR80 cell proliferation was inhibited by treatment with PEDF *in vitro*.

MTS proliferation assay was performed to examine the effects of PEDF on osteosarcoma cell proliferation *in vitro*. Proliferation of OPGR80 cells was inhibited by PEDF treatment in a concentration-dependent manner. Across the range of tested concentrations (1.56, 3.125, 6.25, 12.5, 25, 50, 100 nM), maximal inhibition was achieved by 100 nM PEDF treatment, with a 28.7 ± 3.56% reduction in absorption ratio demonstrated (*p* < 0.01) (Figure 1).

### Luciferase expression by OPGR80 osteosarcoma cells

**Figure 2:**
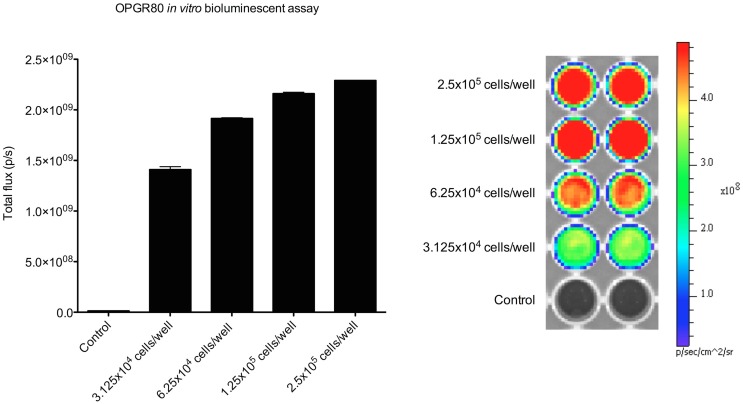
***In vitro* bioluminescent assay**. Luciferase expression by the OPGR80 cell line was confirmed by *in vitro* bioluminescent assay prior to *in vitro* use.

Prior to intra-tibial injection, the luciferase expression by the OPGR80 cell line was determined. All seeded wells were seen to contain luciferase-expressing cells on bioluminescent assay. All concentrations of OPGR80 cells seeded were statistically distinguishable from the control wells and each other (*p* < 0.0001) (Figure 2).

### Primary tumor growth by bioluminescent assay and caliper measurement

From day 3 after intra-tibial injection an exponential pattern of growth of the primary tumor was observed. At day 13, a significant increase in primary tumor total flux was observed (*p* < 0.05). Beyond day 13, a 7-day lapse between serial bioluminescent assays was required to show significant tumor growth by primary tumor total flux.

**Figure 3:**
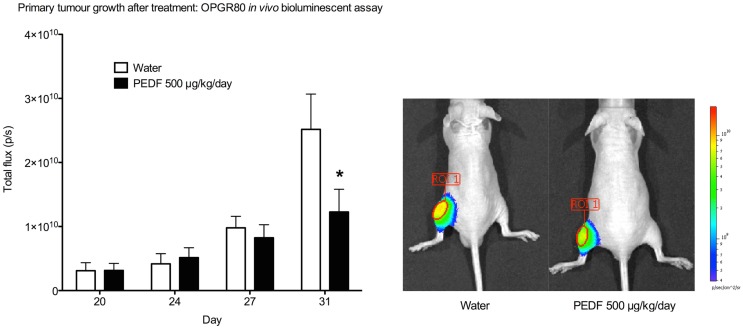
**Primary tumor growth after treatment by *in vivo* bioluminescent assay**. Total flux (p/s) for the primary tumor region of interest (ROI 1) was determined at day 20, 24, 27, and 31. Primary tumor total flux is reduced by PEDF treatment at day 31 (*p < 0.001). Photomicrographs taken at day 31 are shown.

Treatment with PEDF was started at day 20. Average tumor volume at day 20 was 7.50 ± 1.48mm^3^. Treatment with PEDF caused a significant reduction in tumor growth by day 31 of the study when compared to the group receiving sterile water. Primary tumor total flux was reduced by 51.21% (*p* < 0.001) at day 31 (Figure 3). Tumor volume, as calculated from anterioposterior and lateral dimensions, from day 20 to the study endpoint, showed no significant difference between treatment groups (Table 1).

**Table 1:** **Tumor volumes (PEDF 500 μg/kg/day versus water)**.

Day	Water (mm^3^)	PEDF 500 μg/kg/day (mm^3^)	Difference (mm^3^)	95% CI of diff. (mm^3^)	*t*	*p* Value
20	8.555	4.943	−3.611	−20.42 to 13.20	0.6587	*p* > 0.05
24	22.32	20.22	−2.094	−16.62 to 12.43	0.4421	*p* > 0.05
27	21.92	17.15	−4.772	−19.30 to 9.752	1.007	*p* > 0.05
31	24.22	24.35	0.1317	−14.39 to 14.66	0.02780	*p* > 0.05

### Primary tumor growth by PET imaging

**Figure 4:**
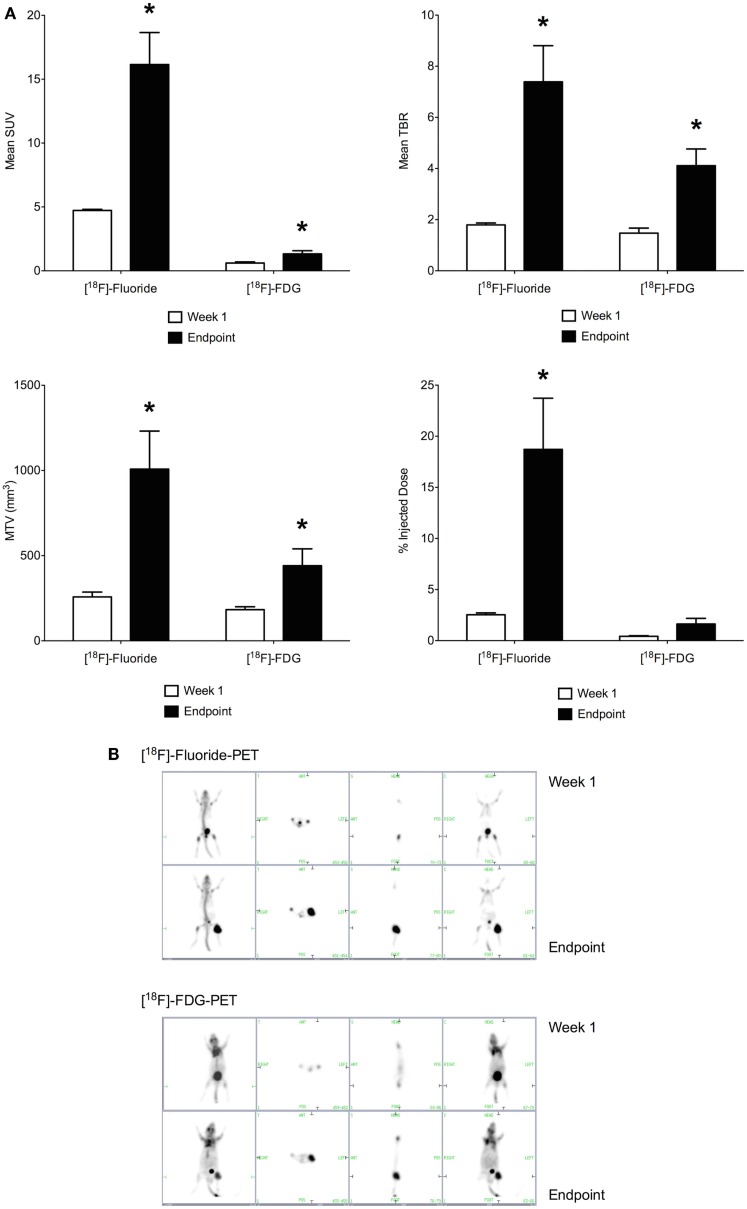
**(A)** Positron-emission tomography parameters obtained 1 week following intra-tibial injection and at the study endpoint (SUV, standardized uptake value; TBR, tumor to background ratio; MTV, metabolic tumor volume; *p < 0.05). [^18^F]-Fluoride SUV was the most sensitive parameter for detecting early stage disease. Between week 1 and the study endpoint, the sevenfold increase in [^18^F]-Fluoride % ID was the greatest, making it the most sensitive marker of disease progression. **(B)** [^18^F]-Fluoride and [^18^F]-FDG-PET imaging were performed at the end of week 1 and the study endpoint. Images show increased uptake of the PET tracer in the left hind limb of the mouse. Pulmonary metastatic disease was not detectable on PET imaging.

Imaging with [^18^F]-Fluoride and [^18^F]-FDG were performed on sequential days, at days 8 and 9 of the study and at days 32 and 33. Maximum standardized uptake value (SUV), TBR, percentage of injected dose (% ID), and MTV were calculated. [^18^F]-Fluoride SUV was the most sensitive parameter for detecting early stage disease in the OPGR80 orthotopic osteosarcoma model, with an average [^18^F]-Fluoride SUV reading of 4.723, 1 week after intra-tibial injection. Taking arbitrary cut-offs of 2.0 and 3.0 for SUV and TBR, respectively, other readings obtained at week 1 were not of sufficient magnitude to indicate malignancy (Figures 4A,B).

Between week 1 and the study endpoint, the sevenfold increase in [^18^F]-Fluoride % ID was the greatest, making it the most sensitive marker of disease progression. When results for [^18^F]-Fluoride and [^18^F]-FDG are compared, the increases in SUV, TBR, MTV, and % ID demonstrated for [^18^F]-Fluoride were of greater magnitude than for [^18^F]-FDG (Figures 4A,B). Application of PET imaging to examine the potential therapeutic effects of PEDF showed that treatment with PEDF did not reduce primary tumor progression.

### Pulmonary metastases by bioluminescent assay and PET imaging

*In vivo* bioluminescent assays were performed to monitor both primary tumor and pulmonary metastases. Intrinsic dynamic range limitations associated with the Xenogen IVIS and Living Image software meant that imaging of primary tumor and pulmonary metastases simultaneously was not possible. Shielding was applied to the hind limbs of mice before imaging of the thorax. This allowed pulmonary metastatic disease to be identified, without interference from the larger hind limb tumors. Isolated images of pulmonary metastases were collected at days 27 and 31 of the study, although metastatic spread at an earlier time-point cannot be ruled out.

**Figure 5:**
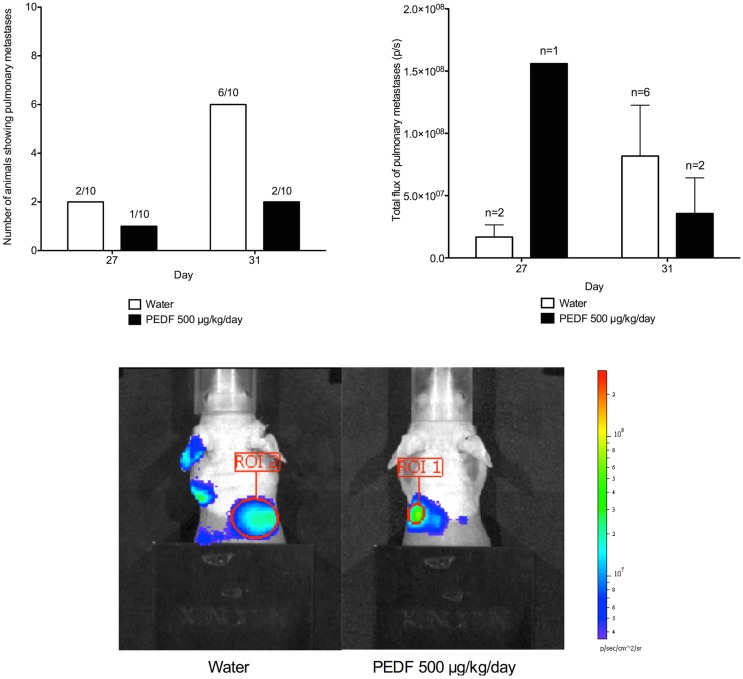
**Detection of pulmonary metastases by *in vivo* bioluminescent assay**. Shielding was applied to the hind limbs of mice before imaging of the thorax. This allowed pulmonary metastatic disease to be identified. Total flux (p/s) for the region of interest (ROI) was determined at day 27 and 31. Photomicrographs taken at day 31 are shown. Treatment with PEDF did not affect the mean burden of pulmonary metastases in these animals.

At day 27 of the study, a total of 16 mice showed pulmonary metastases on *in vivo* bioluminescent assay. By day 31, pulmonary metastatic disease could be identified in a further seven mice. At day 31 of the study, six mice receiving water as control showed evidence of pulmonary metastatic disease compared to two mice receiving PEDF. For those animals that did possess metastases at day 27 and 31, the mean total flux for pulmonary metastases was determined. Treatment with PEDF did not affect the mean burden of pulmonary metastases in these animals (Figure 5).

*In vivo* bioluminescent assays for the identification of pulmonary metastases were performed for all animals on the day prior to PET imaging. The [^18^F]-Fluoride and [^18^F]-FDG-PET scans obtained for animals with known pulmonary metastases were examined. The resolution of the PET images obtained would not allow for reliable identification of pulmonary lesions in any animal.

### Post-mortem assessment of tumor by micro-computed tomography

**Figure 6:**
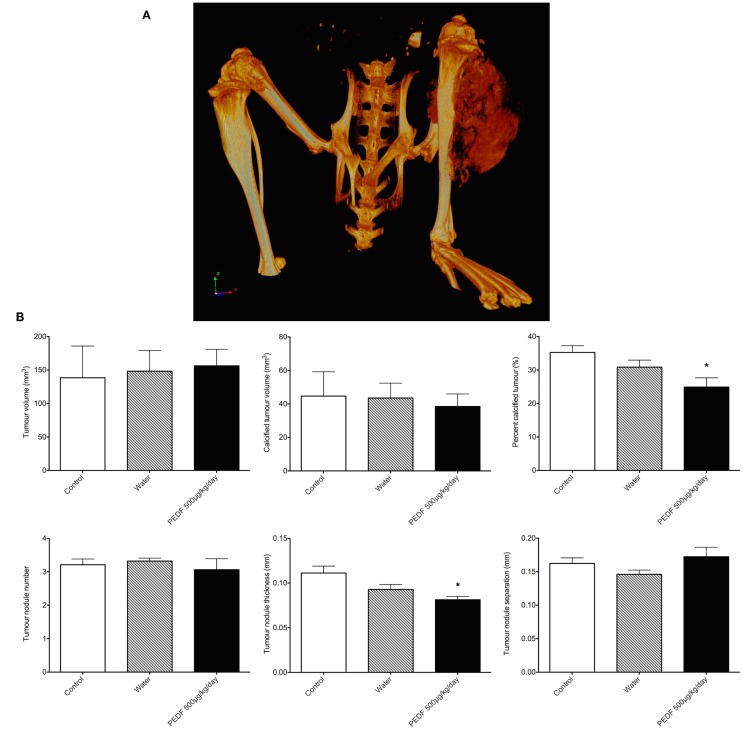
**(A)** Rendered 3D reconstruction obtained by micro-computed tomography. The tumor attached to the proximal tibia is a heterogeneously mineralized mass. **(B)** Primary tumor characteristics by micro-computed tomography. Heterogeneous radio-density of the OPGR80 orthotopic tumor allowed demarcation and segmentation of the tumor as a region of interest (ROI). Tumor volume, radio-dense tumor volume, percentage radio-dense tumor volume, tumor nodule number, thickness, and separation were determined. Animals treated with PEDF (500 μg/kg/day) exhibited tumors that were less radio-dense when compared to the control group.

Heterogeneous radio-density of the OPGR80 orthotopic osteosarcoma allowed demarcation and segmentation of the tumor as a ROI (Figure 6A). Animals treated with PEDF exhibited tumors that were less radio-dense when compared to the control group. PEDF treated animals possessed tumors that were 24.90 ± 2.77% mineralized compared to 35.27 ± 1.99% for the control group (*p* < 0.05). The PEDF treated group possessed tumors with reduced tumor nodule thickness. Mean tumor nodule thickness was reduced by 27% for PEDF treated animals (*p* < 0.01). There was no significant difference between treatment groups for tumor volume, mineralized tumor volume, tumor nodule number, and tumor nodule separation (Figure 6B).

### Histological assessment

#### Primary Tumors

**Figure 7:**
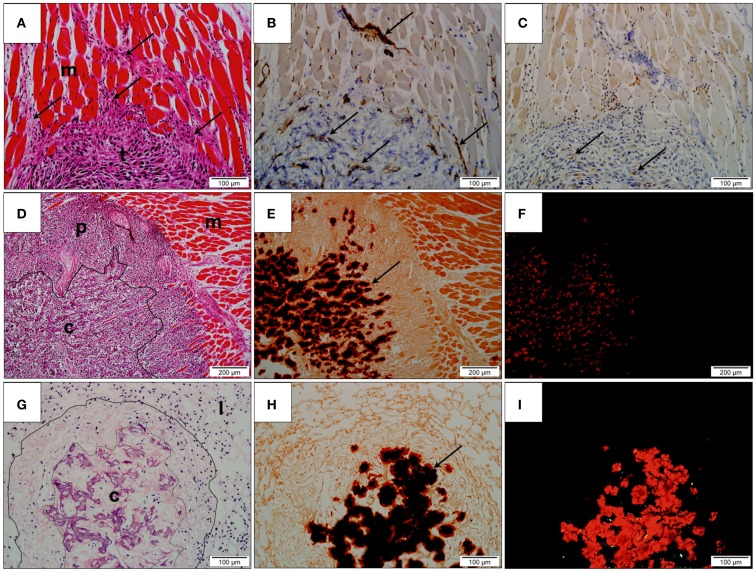
**Histological assessment**. **(A)** Hematoxylin and eosin (H&E) stained section of undifferentiated primary tumor (t). Tumor cells (arrows) are observed infiltrating the adjacent skeletal muscle (m); **(B)** Immunohistochemistry for CD31 showing positively stained endothelium (arrows), within both muscle and tumor; **(C)** Immunohistochemistry for VEGF-1 showing positively stained cells within the peripheral region of the tumor (arrows); **(D)** H&E stained section of tumor and adjacent skeletal muscle (m). The tumor consists of a highly cellular peripheral zone (p) and a matrix-producing central zone (c); **(E)** Alizarin Red S stained tumor identifying the presence of deposited calcium as dark red precipitate (arrow) within the central zone; **(F)** Alizarin Red S stained tumor under polarized light confirms the presence of calcium within tissue evident here as bright red precipitate; **(G)** H&E stained lung tissue (l) with metastatic lesion (contained within solid line). The metastasis possesses a matrix-producing central zone (c); **(H)** Alizarin Red S stained section showing the presence of calcium as dark red precipitate in the central zone (arrow); **(I)** Alizarin Red S stained lung tissue under polarized light. Calcified regions are evident as bright red precipitate.

Hematoxylin and eosin stained sections demonstrated tumor tissue consisting of pleomorphic spindle-shaped cells producing an eosinophilic matrix. Variable degrees of tumor differentiation were seen both within individual samples and across treatment groups. Some tumors consisted solely of pleomorphic spindle cells, while others showed a peripheral zone of invading malignant cells surrounding a central zone of increased matrix production. All sections showed cells at the margin of the tumors were observed infiltrating between striated skeletal muscles. Cells were also observed invading neurovascular structures (Figure 7A). No distinct tumor morphological patterns have been observed between the treatment groups.

The potential anti-angiogenic effect of PEDF on tumor vascularization was examined by identifying CD31-positive vessels and regions of VEGF-A production in the stained tissue. Peripheral regions consisting primarily of invading malignant spindle-shaped cells possessed a fine network of CD31-positive vessels that were closely associated with VEGF-A-producing stromal cells. Although individual CD31-positive inflammatory cells were evident in the surroundings of the central calcified zone, the CD31-positive vessels, and VEGF-A positive cells were largely absent from the central calcified zone of the tumors (Figures 7B,C).

Alizarin Red S staining of tumor tissue showed the central eosinophilic regions of tumor to be birefringent under polarized light indicating that the region was largely calcified (Figures 7D–F). Quantification of Alizarin Red S-calcium complex showed that tumors obtained from control animals contained 36.68 ± 6.273% calcified material, whereas tumors treated with 500 μg/kg/day PEDF contained 36.94 ± 2.099% of calcified material. No statistically significant difference in the degree of tumor calcification was observed between treatment groups.

#### Pulmonary Metastases

Pulmonary metastases were observed as clusters of tumor cells within the native alveolar architecture of the lung (Figure 7G). As was observed for orthotopic primary tumors, variable degrees of matrix production and calcification were demonstrated. At one end of the spectrum, metastatic lesions consisted primarily of tumor cells with minimal matrix production, while at the other end of the spectrum, metastases consisted of a highly cellular peripheral zone surrounding a region of matrix production and calcification. Positive staining with Alizarin Red S that was birefringent under polarized light confirmed this (Figures 7H–I).

Quantification of lung metastases in a maximal en face cross-sectional area showed no statistical difference between treatment groups based on either number of metastatic lesions per section or the cross-sectional area of these lesions. For animals treated with PEDF (500 μg/kg/day), 2.833 ± 1.138 metastases were counted per lung section compared to 2.167 ± 1.108 metastases for the control group. The mean cross-sectional area of micrometastatic lesions was 1.856 × 10^6^ ± 4.405 × 10^5^ μm^2^ for control animals, while animals treated with PEDF possessed pulmonary micrometastases measuring 2.08 × 10^6^ ± 1.08 × 10^6^ μm^2^.

Quantification of Alizarin Red S–calcium containing tumor tissue in pulmonary metastases showed that control animals lesions contained 14.9 ± 4.9% calcified tissue compared to 14.09 ± 3.018% for animals treated with PEDF (500 μg/kg/day). This difference was not statistically significant.

Pulmonary micrometastases are vascularized by an irregular and delicate network of CD31-positive vessels. Similarly to primary orthotopic tumors, the vascularization occurred primarily in the highly cellular peripheral zones of the tumor mass. VEGF-A producing cells, that on histological examination appear to be inflammatory cells, were confined to periphery of the calcified region. Significant inflammatory cell population was observed surrounding the lung metastases, whereas in primary orthotopic tumors, the inflammatory cells were scattered throughout the tumor tissue and were particularly prominent at the invading front of the tumor tissue.

## Discussion

In this study, we report a preclinical murine model of orthotopic osteosarcoma employing the transgenic OPGR80 osteosarcoma cell line. The model is characterized by the deposition of calcified material within the orthotopic tumor tissue as well as in pulmonary metastases. These features make it distinct from previously used models. Earlier *in vivo* studies used the SaOS-2 human osteosarcoma cell line, which gave rise to undifferentiated tumors consisting of pleomorphic spindle cells with little extracellular matrix (6, 8, 10, 11). In this study, we sought to improve the replication of the human condition by the model, and to assess primary and metastatic osteosarcoma through the implementation of advanced imaging techniques.

Pigment epithelium-derived factor inhibited proliferation of OPGR80 osteosarcoma cells *in vitro*. The viability of OPGR80 cells treated 100 nM PEDF was reduced by 28.7 ± 3.56%. When SaOS-2 and SJSA-1 human osteosarcoma cell lines were treated with 100 nM PEDF, the viability of these cells lines was reduced by 13.8 and 36.4%, respectively (6). Reduced osteosarcoma cell proliferation with PEDF treatment has previously been attributed to the induction of osteosarcoma cell apoptosis and the inhibition of cell cycle progression (12).

No previous studies have allowed comparison of bioluminescent imaging and PET imaging in a murine model of orthotopic osteosarcoma. Studies using the SaOS-2 human osteosarcoma cell line, without a luciferase reporter gene, were unable to confirm tumor establishment in the tibia until day 20 after intra-tibial inoculation (6, 8). In the current study, *in vivo* bioluminescent assays performed at day 3 after intra-tibial inoculation of the OPGR80 osteosarcoma cell line confirmed that all mice possessed biologically active tumor cells in the left hindlimb. PET imaging at day 9, using the [^18^F]-FDG radioactive tracer, did not allow early identification of disease. [^18^F]-Fluoride SUV was the only PET parameter that indicated early disease, with an average [^18^F]-Fluoride SUV reading of 4.723 at day 8. Taking arbitrary cut-offs of 2.0 and 3.0 for SUV and TBR, respectively, other parameters at days 8 and 9 were not of sufficient magnitude to indicate malignancy. The early identification of biologically active tumor cells by *in vivo* bioluminescent imaging or [^18^F]-Fluoride SUV is beneficial when closely monitoring disease progression in an animal model such as this.

Treatment with PEDF caused a significant reduction in tumor growth by day 31 of the study by *in vivo* bioluminescent assay. Primary tumor total flux was reduced by 51.21% (*p* < 0.001) at day 31. Reduced tumor growth by *in vivo* bioluminescent assay and absence of a therapeutic effect demonstrated on digital caliper measurement, in addition to the much earlier confirmation of tumor establishment in bone, suggests that the use of serial bioluminescent assays is a sensitive method for detecting and monitoring osteosarcoma progression in the OPGR80 model.

The murine model of orthotopic osteosarcoma reported here is the first model to utilize PET imaging, with [^18^F]-Fluoride and [^18^F]-FDG radioactive tracers, to evaluate PEDF therapy. While treatment with PEDF did not reduce primary tumor progression, a number of findings warrant discussion as the model is likely to be used further for the assessment of other novel anti-osteosarcoma agents. [^18^F]-Fluoride-PET was more sensitive than [^18^F]-FDG-PET for detecting early disease in the OPGR80 model. Furthermore, while both [^18^F]-Fluoride-PET and [^18^F]-FDG-PET showed statistically significant increases in PET parameters between week 1 and the study endpoint, the sevenfold increase in [^18^F]-Fluoride % ID was the greatest, making it the most sensitive marker of disease progression. Osteosarcoma is a tumor characterized by the production of osteoid, which becomes mineralized in better-differentiated tumors. The OPGR80 cell line used in the study gave rise to a model that is heavily characterized by calcification of both primary and metastatic lesions. The increased sensitivity of [^18^F]-Fluoride-PET is in line with this characteristic.

Post-mortem micro-computed tomography showed that tumors treated with PEDF were less radio-dense than those of control animals, an attribute that may be accounted for by reduced tumor nodule thickness. However, post-mortem histological assessment of tumor calcification by Alizarin Red S staining did not show a statistical difference between treatment groups. A more comprehensive study utilizing stereo-logical 3D volumetric counting may be of benefit. It is important to note here that the analysis of radio-density on micro-computed tomography was performed by segmentation of 3D volumetric reconstructions, while percent Alizarin Red S staining was obtained from representative tissue sections.

The strength of the current study lies in its use of multiple modalities for the assessment of primary and metastatic osteosarcoma progression. Indeed, a similar approach is used in the human condition for evaluation of primary tumors and staging. Radiographs, CT, MRI, bone scan, and PET are all used clinically to characterize both primary and metastatic disease. PET in particular plays a role in staging, prognostication, and may guide surgical treatment. For FDG-avid sarcomas, PET may be used to guide biopsy of regions with the most intense uptake, which are likely to represent the highest grade of disease (13, 14). PET is also useful for the diagnosis of sarcomatous chance in the setting of neurofibromatosis and chondromatosis. When FDG avidity is present at the time of diagnosis, a baseline is obtained for assessing the response to adjuvant therapy, an indicator of prognosis (15). Additionally, when a poor response to adjuvant therapy is seen, wider surgical margins may be considered. In the current study, serial PET evaluation demonstrated disease progression in the orthotopic murine model and was unable to identify metastatic lesions. While the purpose of this study was not to clarify the role of PET in staging and treating osteosarcoma, the results here provide proof of principle that [^18^F]-FDG- or [^18^F]-Fluoride-PET may be useful for evaluating novel treatment agents using the orthotopic murine model of osteosarcoma. In the future, advanced imaging and targeted probes for osteosarcoma may assist with staging and treating human disease.

Limitations of the study include the use of a transgenic murine osteosarcoma cell line rather than a human cell line, and the inability to compare systemic PEDF treatment to a mainstay chemotherapeutic. While a therapeutic response to PEDF was demonstrated on *in vivo* bioluminescent imaging the significance of this finding cannot be concluded without reference to an agent with an established therapeutic effect.

## Conclusion

This study demonstrates the value of advanced imaging techniques in the study of tumor growth, invasion, and metastasis. *In vivo* bioluminescent imaging and [^18^F]-Fluoride-PET allow early identification of disease in the OPGR80 murine model of orthotopic osteosarcoma. Both [^18^F]-Fluoride-PET and [^18^F]-FDG-PET have demonstrated disease progression. High uptake of [^18^F]-Fluoride in tumor provides a greater potential for detection of signal modulation by therapeutic intervention.

## Conflict of Interest Statement

The authors declare that the research was conducted in the absence of any commercial or financial relationships that could be construed as a potential conflict of interest.
